# Woman with Pregnancy and Lactation-Associated Osteoporosis (PLO)

**DOI:** 10.1155/2020/8836583

**Published:** 2020-11-12

**Authors:** Nasrin Bazgir, Elham Shafiei, Neda Hashemi, Hassan Nourmohamadi

**Affiliations:** ^1^Department of Rheumatology, Medical School, Ilam University of Medical Sciences, Ilam, Iran; ^2^Clinical Research Development Unit, Shahid Mostafa Khomeini Hospital, Ilam University of Medical Sciences, Ilam, Iran; ^3^Rasoul Akram Hospital, Iran University of Medical Sciences, Tehran, Iran; ^4^Department of internal medicine, Shahid Mostafa Khomeini Hospital, Ilam University of Medical Sciences, Ilam, Iran

## Abstract

Osteoporosis is a disease known to reduce bone density and to damage bone microarchitecture leading to increased fracture risk. Osteoporosis is one of the most common diseases among the middle aged and elderly people that impose high costs on the community. So far, despite rare cases of pregnancy and lactation-associated osteoporosis (PLO) reported in Iran, it can be treated with accurate diagnosis. *Patient Introduction*. A 24-year-old woman was referred to the outpatient rheumatologic clinic after the cesarean section during the first pregnancy with severe back pain. In the thoracolumbar radiographs, a loss of vertebral height in the T11 to L5 vertebra was recognized. Other complaints were abdominal pain and anemia. In order to evaluate the gastrointestinal disease and celiac, the patient underwent gastrointestinal and hematologic workup. Ultimately, secondary causes of the osteoporosis were excluded. Based on the patient's clinical course, imaging finding, and exclusion of other causes of osteoporosis, the patient was diagnosed with PLO. *Conclusion*. Clinicians should be aware of PLO as rare complication of pregnancy. The situation should be particularly considered in females offering from new onset back pain in the third trimester of pregnancy or breastfeeding period.

## 1. Introduction

Osteoporosis is described by a decrease in bone density, and bone microarchitecture quality is lowered [1].

Osteoporosis usually develops gradually with no symptoms. It is discovered only when a fracture occurs [2].

The most common signs of osteoporosis contain fracture and pain. Frequency of pregnancy and lactation-associated osteoporosis (PLO) is valued to be 4–8 individuals for every one million women [3, 4].

During pregnancy or breastfeeding, the healthy females are rarely affected by the PLO. Pregnancy has no risk factors for osteoporotic fractures [5, 6] arising commonly at the thoracolumbar vertebral [7–9]. The PLO was identified almost in 70% of the women through their first pregnancy [10].

During pregnancy, mainly, the third trimester of the fetal bone skeleton necessities totalling 30 g of calcium (the equivalent of 250–300 mg daily calcium) should be provided from the mother [11].

Lactating women drop daily a usual amount of 210 mg of calcium during breastfeeding. This means that after the first six months of breastfeeding, every lactating woman loses a total of calcium four times more than that of pregnancy period [12].

As far as the PLO is concerned, both the increased weight-bearing and lordotic posture in pregnant women would lead to low bone mass. Also, skeletal fragility may lead to spine fractures just because of a low trauma [13].

## 2. Patient Introduction

The patient is a 24-year-old woman complaining of severe back pain. The patient was under medical treatment, but the nonsteroidal anti-inflammatory drugs (NSAIDs) along with physiotherapy had nothing to alleviate the pain. In the thoracolumbar radiographs, a loss of vertebral height in the T11-L5 was detected.

**Figure 1:**
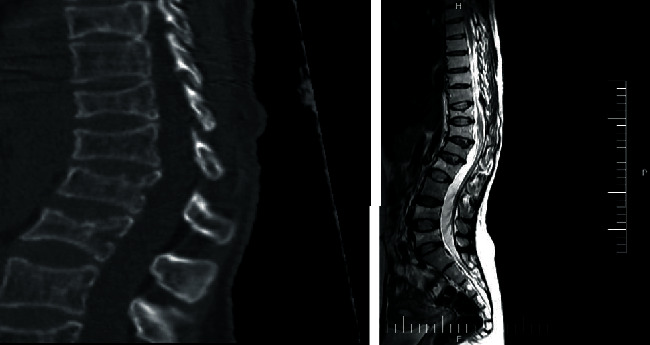
The result of a patient's imaging indicating fracture in the (T11-L5) vertebral.

In the same area, multiple compression fractures were illustrated through (MRI) (Figure 1). The dual-energy X-ray absorptiometry (Hologic Discovery system) was applied to quantify the bone mineral density (BMD) at the vertebral spine and the hip. The BMD confirmed the osteoporosis. In addition, the patient was suffering from abdominal pain and anemia. Gastrointestinal examinations and related tests were normal as reported by the gastroenterologist. Only anemia due to iron deficiency was reported by an oncologist for further blood tests. Other examinations were healthy.

The patient was advised to discontinue lactation as soon as PLO was established. Immediately, we prescribed calcium 500 mg per day, vitamin D3 50000 every 2 weeks, and teriparatide 20 *μ*g/d. An improvement in the patient's pain and movement was seen three weeks after the therapy (Table 1). As far as the spinal and hip BMD are concerned, the control BMD was shown to be increased 18 months after the treatment. We were faced with no novel fractures during the treatment (Table 2).

## 3. Discussion and Conclusion

Although osteoporosis happens at middle age and senile population, osteoporosis is uncommon in pregnancy.

Primiparous women are affected by the PLO. Risk factors of PLO included fractures and PLO in the first degree families, osteoporosis history, vitamin D deficiency, low body mass index (BMI), inadequate intake of calcium, low physical activity, therapy with anticoagulants and ppi, elevated parathyroid hormone-related protein (PTHrP) and high rates of bone turnover, and finally smoking [14, 15]. Disabled LBP and reduced height resulted from fractures in the vertebral osteoporosis in the third trimester or in the lactation period. Termination of breastfeeding, bisphosphonates prescription, and supplementation of calcium and vitamin D are considered as the main frequently used therapies for PLO.

One of the rare causes of back pain during pregnancy is the PLO which occurs mostly when the woman is pregnant for the first time. Occasionally, back pain shows itself in the third trimester or when the woman has delivered. Another significant indicator of the PLO is height loss. As reported, both the lower thoracic and the lumbar spine are known as the two most affected sites by fractures. It is evident that the loss of the BMD in the spine is more common and severe compared to the other parts of the skeleton [16].

Increased bone turnover is quite obvious during pregnancy and lactation. Maternal bones deliver to the fetus 110-120 mg/kg/d of calcium, and during the third trimester, the mother experiences a decreased BMD in the range of 2-4%. Lactation period is attributed to higher calcium loss about 300 mg/day compared to the pregnancy period. The breastfeeding women lose about 1-3% of BMD per month. As far as bone metabolism is concerned, prolactin and the parathyroid-related peptide (PTHrP) are the main modulators during pregnancy and breastfeeding. Prolactin secretion is stimulated by lactation which in turn decreases the gonadotropin production, and this leads ultimately to the secretion of lower levels of estradiol. Also, the production of PTHRP is increased by prolactin and, on the other hand, increased the PTHRP along with the low estradiol making synergistically the bone resorption.

## Figures and Tables

**Table 1:** Laboratory markers before and 18 months after treatment.

Index	Before treatment	After treatment	Normal range
25OH vitamin D (ng/ml)	29	40	10-30
Phosphorus (mg/dl)	2.7	3.3	2.5-4.5
Intact PTH (pg/ml)	18	12	10-66
24 h urine calcium (mg/dl)	124	180	100-250
Calcium (mg/dl)	8.5	8.9	8.5-10.5
ALP (IU/l)	306	319	200-350

**Table 2:** BMD before and 18 months after treatment.

	L (1–4)		Femur neck	
	Before treatment	After treatment	Before treatment	After treatment
T-score	-3.6	-1.8	-3.1	-1.9
Z-score	-3.3	-1.8	-3	-2
BMD (g/cm^2^)	0.540	0.580	0.510	0.560

BMD: bone mineral densitometry.
